# Modelling the Cost-Effectiveness of a Placental Malaria Vaccine in Sub-Saharan Africa

**DOI:** 10.3390/vaccines14050378

**Published:** 2026-04-23

**Authors:** Jobiba Chinkhumba, Lucinda Manda-Taylor, Flavia D’Alessio, Mwayiwawo Madanitsa

**Affiliations:** 1 Department of Health Systems and Policy, School of Global and Public Health, Kamuzu University of Health Sciences, Blantyre P/Bag 360, Malawi; 2 Department of Bioethics and Behavioural Social Sciences, School of Global and Public Health, Kamuzu University of Health Sciences, Blantyre P/Bag 360, Malawi; mandal@kuhes.ac.mw; 3 European Vaccine Initiative, Im Neuenheimer Feld 515, 69120 Heidelberg, Germany; flavia.dalessio@euvaccine.eu; 4 Academy of Medical Sciences, Malawi University of Science and Technology, Thyolo P.O. Box 5196, Malawi; mmadanitsa@must.ac.mw

**Keywords:** cost-effectiveness analysis, sub-Saharan Africa, Placental malaria vaccine

## Abstract

**Introduction:** Placental malaria increases the risk of adverse birth outcomes. Current preventive measures are undermined by poor coverage, growing resistance to chemo-preventive and therapeutic drugs, and vector eliminating insecticides. Candidate placental malaria (PM) vaccines (PAMVAC and PRIMVAC) have shown safety and immunogenicity in Phase I trials, but empirical evidence on their potential population-level value is lacking. This study modelled the expected cost-effectiveness of a PM vaccine administered before pregnancy. **Methods:** A decision-analytic model compared two strategies from the provider’s perspective: vaccinating women of childbearing age versus no vaccination. The model incorporated gravidity-specific risks of PM, neonatal mortality and the malaria attributable fractions from the literature. Since the efficacy of a PM vaccine for malaria prevention is unknown, we assumed a 40% efficacy and varied this estimate widely in sensitivity analyses. Primary outcomes were incremental cost-effectiveness ratios (ICERs) per perinatal disability adjusted life years (DALYs) averted. Baseline, best-case, and worst-case scenarios were analysed. One-way and probabilistic sensitivity analyses were used to assess parameter uncertainty. Cost-effectiveness was defined as an ICER below half of sub- Saharan Africa’s 2025 GDP per capita ($1556). **Results:** The vaccine was most cost-effective among primigravidae. Under baseline assumptions (40% efficacy; 30% uptake; $5 dose price), the ICER was $321 per perinatal DALY averted for primigravidae versus $4444 for multigravidae. Best-case assumptions further improved cost-effectiveness ($225 vs. $3148). Sensitivity analyses showed robust cost-effectiveness for primigravidae across all plausible parameter ranges, while ICERs in multigravidae were highly sensitive to programme costs and vaccine efficacy. Cost-effectiveness acceptability curves demonstrated that vaccination becomes favourable for primigravidae at relatively low willingness-to-pay thresholds. **Conclusions:** A placental malaria vaccine delivered before pregnancy has high potential to be cost-effective in endemic areas when targeted to protect primigravidae. These findings support prioritised deployment strategies and highlight the value of early economic modelling to inform vaccine development and policy planning.

## 1. Introduction

Malaria in pregnancy (MiP) remains a significant public health concern, particularly in sub-Saharan Africa (SSA), where the burden is concentrated [1] and *P. falciparum* infection is predominant. Of the estimated 36 million pregnancies that occurred in the region in 2023, about 12.4 million (34%) were exposed to malaria infection [2]. The detrimental impacts of malaria during pregnancy not only affect the health of the mother but also have profound consequences for the foetus. These include an increased risk of low birth weight, preterm delivery, stillbirths and neonatal deaths [3].

Despite the availability of cost-effective preventive measures, including intermittent preventive treatment with sulfadoxine-pyrimethamine (IPTp-SP), insecticide-treated nets (ITNs) and indoor residual spraying (IRS), their overall impact remains constrained by widespread parasite resistance to SP [4], mosquito resistance to insecticides [5], and inadequate coverage of at-risk populations. Recent estimates indicate that the average uptake of IPTp-SP among pregnant women across SSA countries was 30.7%, with significant variation between countries [6]. The pooled prevalence of ITN use among pregnant women was 51.6% [7], both of which are below the 80% operational goal by the World Health Organization (WHO) [8].

Recent advances in the development of placental malaria vaccine candidates targeting *Plasmodium falciparum* offer promising novel tools to provide additional protection to at-risk women and reduce the incidence of MiP-related adverse outcomes [9]. The PAMVAC and PRIMVAC PM vaccine candidates have successfully completed phase I clinical studies in Europe and Africa [10,11]. Plans are underway to evaluate an optimised version of PAMVAC coupled to capsid virus like particles (PAMVAC-cVLP) and to conduct a PRIMVAC phase II clinical trial to evaluate the vaccine’s efficacy. However, clinical trials assessing the impact of MiP interventions can be costly and time-consuming, often requiring a few years of longitudinal follow-up [12]. Although not a substitute for clinical trials and still infrequently applied, modelling provides a valuable approach to assessing the potential value and impact of new health interventions [13]. This is particularly relevant in contexts where alternative options already exist, and any new intervention must demonstrate comparative advantage [14]. Growing evidence further underscores the importance of undertaking modelling studies in the early phases of health intervention development [15,16].

This study was part of the European Union-funded ADVANCE VAC4PM project, led by the European Vaccine Initiative (EVI) [17]. We aimed to conduct a Stage 1 economic evaluation of a potential Placental malaria vaccine by developing a decision-analytic model to estimate its expected cost-effectiveness in averting disability-adjusted life years (DALYs) through the prevention of perinatal deaths. We focused on perinatal outcomes, specifically stillbirths and neonatal deaths, because placental malaria has a well-established and substantial impact on adverse birth outcomes [18,19]. These outcomes represent a major and directly attributable burden of disease in this population, and robust data are more readily available to parameterize the model with greater certainty at this stage.

The analysis incorporated key factors, including vaccine efficacy, costs, and uptake among at-risk women of childbearing age in malaria-endemic settings. We conducted a stratified analysis of the expected costs and health benefits of vaccine administration before pregnancy among primigravidae and multigravidae. This allowed us to address a critical policy question: how targeting a gravidity-specific group can inform optimal deployment strategies for a placental malaria vaccine in endemic regions. The findings of this study will contribute to the growing body of evidence needed to inform the development of a placental malaria vaccine and future vaccination strategies aimed at reducing the devastating impact of perinatal deaths and improving the overall health of pregnant women, while also identifying priority outcomes for future empirical studies.

## 2. Methods

We followed the Consolidated Health Economic Evaluation Reporting Standards (CHEERS) guidelines to ensure comprehensive, transparent, and consistent reporting of all methods and results [20].

### 2.1. The Model Structure

The primary decision-analytic model compared two policy strategies reflecting decision-maker options: (a) vaccinating women of childbearing age with a placental malaria (PM) vaccine before pregnancy (intervention), and (b) not offering vaccination (comparator). While policymakers determine vaccine allocation, individual women make autonomous decisions regarding PM vaccine receipt. Consistent with mechanistic maternal immunisation models (e.g., Tdap IgG kinetics and placental transfer), our model assumes that administering the PM vaccine prior to pregnancy allows the development of antibodies able to confer optimal protection against placental malaria once the women become pregnant and are exposed to *P. falciparum* infection [21]. Vaccination before pregnancy would also prevent concerns of potential harm to the newborn in contrast to administration during pregnancy [22]. For both strategies, it was assumed that all pregnant women would also receive standard MiP preventive measures in accordance with regional guidelines, specifically, IPTp-SP and ITNs.

**Figure 1:**
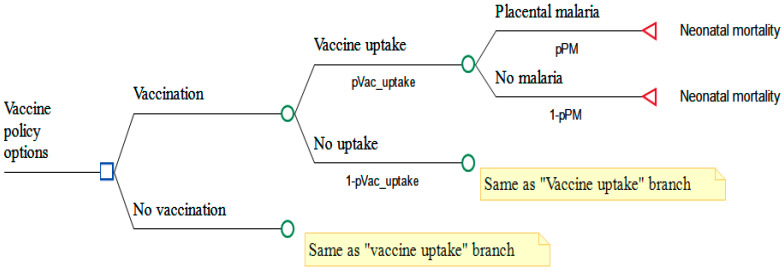
Schema for the placental malaria decision tree model. *p*Vac_uptake = probability of vaccine uptake; *p*PM = probability of Placental malaria. Rectangle = Decision node, circle = chance node, triangle = payoff.

The time horizon included a single full pregnancy episode and incorporated the risk of neonatal mortality, which is strongly associated with placental malaria [18,19]. The model stratified the risk of neonatal mortality based on maternal PM status, i.e., whether or not the mother had placental malaria [1,19]. Neonatal death was then assigned conditionally on this status. To estimate the outcome of interest (perinatal deaths), neonatal deaths were adjusted to include expected corresponding stillbirth losses. The model was run separately for primigravidae (women who are pregnant for the first time) and multigravidae (women who have been pregnant more than once). Figure 1 provides a schematic overview of the decision model. The full model, developed in TreeAge Pro 2025, is available upon request.

### 2.2. Analytical Overview

The decision tree model was used to estimate the expected health outcomes and costs of administering a PM vaccine from the provider’s perspective. By comparing the vaccination strategy to the status quo, the model estimated the additional (incremental) perinatal life-years gained (LYG) from averted perinatal deaths and the corresponding costs of PM vaccination. Life expectancy at birth estimates for sub-Saharan Africa were used to calculate life-years gained [23]. In the baseline scenario, future LYG were discounted at 3% [14]. LYG were converted to DALYs averted, assuming 1 DALY ≈ 1 LYG (ignoring the year lived with disability (YLD) component and disability weight).

To promote generalisability of results, probability estimates for model parameters were derived from published studies conducted in sub-Saharan Africa (Table 1). The mean value of each probability was used as the baseline estimate, while the reported range informed a plausible range for sensitivity analyses. In addition to the baseline scenario, we developed best-case and worst-case scenarios using the most optimistic and most pessimistic values for selected key variables.

Costs were assigned to each strategy, including the PM vaccine cost and administration/delivery costs in the vaccination arm. Incremental cost-effectiveness ratios (ICERs) were calculated as the difference in costs between the two strategies divided by the difference in their health effects, expressed as cost per DALY averted. In line with current recommendations [24], the PM vaccine was considered cost-effective in the sub-Saharan African context if the ICER was below half of the region’s projected 2025 Gross Domestic Product (GDP) per capita, estimated at $1556 [25]. Both deterministic and probabilistic sensitivity analyses (PSA) were conducted to identify key ICER drivers and assess the robustness of the model’s assumptions.

### 2.3. Efficacy of Placental Malaria Vaccine

The efficacy of the PM vaccine for the prevention of placental malaria in pregnant women is not known yet. An underlying assumption of this study is that receipt of the PM vaccine before pregnancy would decrease the risk of placental malaria by a level well above that conferred by current malaria prevention efforts. Although previous studies have demonstrated high efficacy of a general malaria vaccine in preventing peripheral parasitemia when administered before pregnancy to women of childbearing age [26], we conservatively assumed a lower efficacy rate for placental malaria prevention (40%) in our base-case scenario, given that the placental malaria vaccine operates through a different pathophysiological mechanism [27]. We examined efficacy rates as low as 20% during sensitivity analyses.

### 2.4. Clinical Malaria, Placental Malaria and Neonatal Mortality Risks

To determine how the gravidity status could inform optimal PM vaccine deployment strategies in endemic regions, we evaluated two groups of women: primigravidae and multigravidae. The two groups face varying risks for clinical malaria, placental malaria and neonatal mortality [1,19], as protective immunity can be naturally acquired over successive pregnancies. Consequently, primigravidae living in high transmission areas experience a higher incidence of clinical malaria compared to multigravidae [28,29], with a recent reported relative risk (RR) of 2.87 (95% CI: 2.39–3.45) [30]. A modelling study conducted across sub-Saharan Africa, accounting for the intensity of *P. falciparum* transmission, estimated a mean risk of placental malaria in primigravidae women of 25% (range: 20–39%) [31]. To estimate the corresponding placental malaria risk in multigravidae, we applied the primigravidae risk to the RR and then back-calculated the risk of placental malaria in multigravidae.

A recent study found that primigravid women have a significantly higher risk of neonatal mortality compared to multigravid women, with rates of 27.7 versus 17.2 deaths per 1000 live births (*p* < 0.001) [32]. Neonatal mortality risk is further increased by placental malaria. Among primigravidae, the attributable fraction of neonatal mortality due to malaria in pregnancy ranges from 18% to 42% [30,31], while in multigravidae it ranges from 6% to 11% [33,34,35]. These estimates are likely the most representative of the SSA region and were therefore used in our baseline scenario (Table 1). To estimate neonatal mortality attributable to placental malaria, we combined gravidity-specific neonatal mortality rates with their corresponding attributable fractions. Global data reports that the ratio of stillbirths to neonatal deaths ranges from 0.7 to 1.1 [36]. We therefore inflated neonatal deaths using this range to capture the broader effects of the PM vaccine on perinatal (stillbirth + neonatal) losses.

**Table 1:** Parameters used in placental malaria decision model and their sources.

Parameter Name	Baseline	Low	High	Distribution	Source
Costs ($)					
Uncomplicated malaria in pregnancy	4.39	3.61	4.69	Gamma	[37]
Complicated malaria in pregnancy	88.15	63.33	101.49	Gamma	[37]
Vaccine dose	5	2	10	Gamma	[38]/Assumption
Vaccine delivery	22.62	15.17	48.70	Gamma	[39]/Assumption
Vaccine attributes (%)					
Vaccine uptake	30.0	20.0	50.0	Beta	[35]/Assumption
Vaccine efficacy	40.0	20.0	50.0	Beta	Assumption
Risks and Relative risk (RR)					
Risk of PM in primigravida	0.25	0.20	0.39	Beta	[31]
Risk of PM in multigravida	0.09	0.07	0.11	Beta	[30,31]
RR of PM in vaccinated women	0.60	0.80	0.50	Beta	Assumption
Neonatal mortality in primigravida *	27.7	22.16	33.24	Beta	[32]
Neonatal mortality in multigravida *	17.2	13.76	20.64	Beta	[32]
Attributable fraction in primigravida **	0.24	0.18	0.42	Beta	[33,34,35]
Attributable fraction in multigravida **	0.08	0.06	0.11	Beta	[33,35]
Perinatal deaths per neonatal death	1.9	1.7	2.1	Beta	[36]
Life expectancy at birth	65	60	70	Normal	[23]
Others (%)					
Clinical malaria in pregnancy	19	13	26	Beta	[38]
Uncomplicated malaria in pregnancy	90	95	85	Beta	[40]
Discount rate	3	0	10	Beta	[14]

* Per 1000 live births: ** Share of neonatal deaths in the exposed due to malaria.

### 2.5. PM Vaccine Delivery and Uptake Estimates

In sub-Saharan Africa, three principal vaccine delivery models are typically employed [39]: routine health facility delivery through the Expanded Programme on Immunisation (EPI), community or outreach-based delivery, and mass campaign delivery. It was assumed that implementation of a PM vaccine would similarly involve a combination of these strategies. Using human papillomavirus (HPV) vaccine coverage as a proxy, on the premise that PM vaccination targets a similar population, and its introduction would encounter comparable logistical and health system constraints, the current HPV pooled uptake of approximately 30% was adopted as the baseline scenario [41].

### 2.6. Cost Estimates for Malaria in Pregnancy Treatment and PM Vaccines

Cost estimates for MiP treatment were sourced from the published literature. Costing studies often adopt multiple perspectives. A recent multi-country study assessing the economic costs of MiP in four sub-Saharan African countries reported provider costs per episode of uncomplicated and complicated MiP as follows: $4.69 and $101.41 in the Democratic Republic of Congo; $3.61 and $63.33 in Madagascar; $4.68 and $83.70 in Mozambique; and $4.09 and $92.64 in Nigeria [37]. For the baseline scenario, we used the median values of these cost estimates and conducted sensitivity analyses across the reported ranges. Treatment costs were adjusted to reflect that approximately 90% of MiP cases are uncomplicated [40]. All cost figures were adjusted for inflation using World Bank country-specific GDP deflators [42], and results are presented in constant 2025 US dollars.

The cost of malaria vaccines in sub-Saharan Africa varies by vaccine type, procurement mechanisms, and country-specific co-financing arrangements [43]. Two malaria vaccines are currently in use or being introduced: RTS,S/AS01 (Mosquirix), developed by GlaxoSmithKline, and R21/Matrix-M™, produced by the Serum Institute of India. The assumed cost ($5) for a potential PM vaccine was based on current prices of these approved malaria vaccines, which range from $2 to $10 per dose [44,45]. Vaccine delivery cost estimates vary by delivery strategy [46]. To conservatively reflect programme costs for the PM vaccine, e.g., supply chain, delivery, and staff training, and varying operational contexts (e.g., mass campaign delivery versus outreach-based delivery in hard-to-reach or resource-constrained settings), a median delivery cost of $22.62 was used in the baseline scenario [47], again, using HPV as a proxy.

### 2.7. Sensitivity Analyses and Parameter Uncertainty

The impact of individual model parameters on ICERs was assessed using one-way sensitivity analyses. Each parameter was varied across plausible ranges derived from the literature (Table 1), with the mean/median as the base-case value. The 10 parameters with the most significant influence on ICERs were subsequently included in a probabilistic sensitivity analysis (PSA), with appropriate probability distributions assigned based on parameter type: gamma distributions for cost variables, normal distributions for life years gained (LYG), and beta distributions for service utilisation and risk parameters [48]. Parameter bounds were calculated using the methods of moments. To account for uncertainty, we conducted parametric bootstrapping with 5000 iterations, propagating variation through the model. Results are presented as cost-effectiveness acceptability curves (CEACs). CEACs illustrate the probability that an intervention is cost-effective compared to the comparator at varying levels of willingness-to-pay [49].

### 2.8. Model Validation

The model structure, assumptions, and parameter inputs were reviewed by clinical, vaccinology, epidemiology and health economics experts familiar with MiP and vaccine programme implementation in SSA during two workshop presentations (face validity) [50]. Necessary adjustments based on feedback were incorporated to ensure the model reflected real-world decision-making and programmatic realities in the region. Cost per DALY averted were compared to similar CEA studies on malaria prevention during pregnancy, such as IPTp-SP and ITN interventions. Estimates were within ranges reported in the literature, supporting the model’s external validity.

### 2.9. Role of the Funding Source

Funding for this study was provided by the European Union, Horizon Europe (grant number 101057882). The funder had no role in the study design, data collection, analysis, interpretation of data, or the writing of the manuscript. The authors declare no financial or personal conflicts of interest that could inappropriately influence this work.

## 3. Results

The incremental cost-effectiveness ratios (ICERs) for the PM vaccine varied by gravidity group and scenario (Table 2). At baseline, the vaccine was more cost-effective among primigravidae, with an ICER of $321 per perinatal DALY averted, compared to $4444 among multigravidae. In the best-case and worst-case scenarios, we adjusted the most critical variables (vaccine efficacy, cost and uptake) simultaneously. Under the best-case scenario, the ICERs improved to $225 and $3148 per DALY averted for primigravidae and multigravidae, respectively, while the least favourable scenario increased the ICERs to $780 and $10,598. Overall, the PM vaccine demonstrated consistently greater economic value among primigravidae across all scenarios.

**Figure 2:**
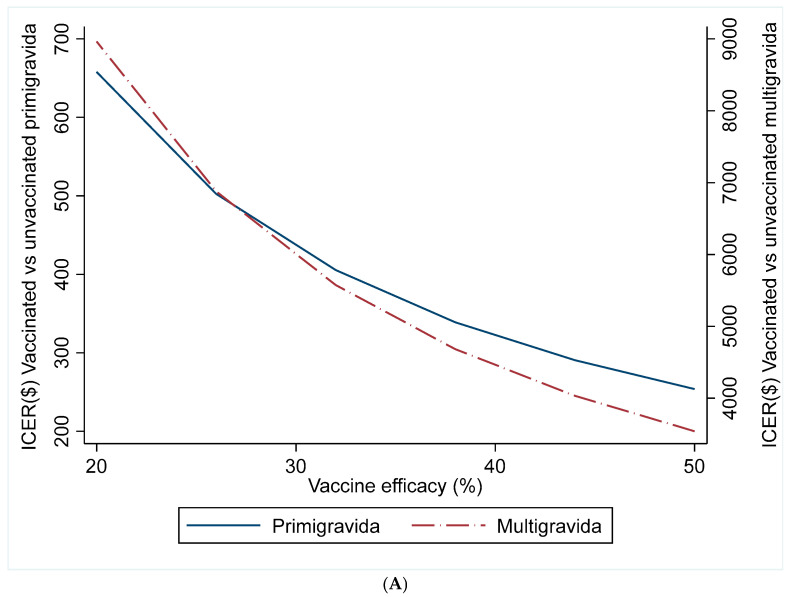

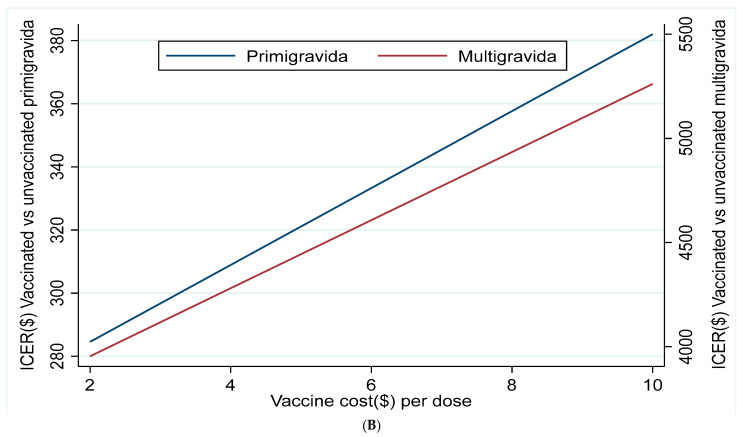
(**A**) Sensitivity analysis of vaccine efficacy on incremental cost-effectiveness ratio. (**B**) Sensitivity analysis of the cost of vaccine per dose on the incremental cost-effectiveness ratio.

Figure 2A Illustrates that the cost-effectiveness of the vaccine varied with its assumed efficacy. At a low efficacy of 20%, the ICERs were $658 per perinatal DALY averted for primigravidae and $8964 per perinatal DALY averted for multigravidae. When efficacy was increased to 50%, the ICERs decreased substantially to $254 per perinatal DALY averted for primigravidae and $3540 per perinatal DALY averted for multigravidae, indicating improved cost-effectiveness with higher vaccine efficacy for both primigravidae and multigravidae. Across all scenarios, vaccination of primigravidae remained more cost-effective than that of multigravidae.

Figure 2B shows the relationship between vaccine cost and the ICER for vaccinating women against placental malaria. As the cost per dose increased from $2 to $10, the ICERs rose for both primigravidae and multigravidae. For $2 per dose, the ICERs were $285 per perinatal DALY averted for primigravidae and $3955 per DALY averted for multigravidae. Increasing the price to $10 per dose raised the ICERs to $382 per perinatal DALY averted for primigravidae and $5261 per DALY averted for multigravidae, indicating that the vaccination becomes less cost-effective at higher vaccine prices. Across all vaccine cost levels, ICERs for primigravidae remain substantially lower than those for multigravidae.

**Figure 3:**
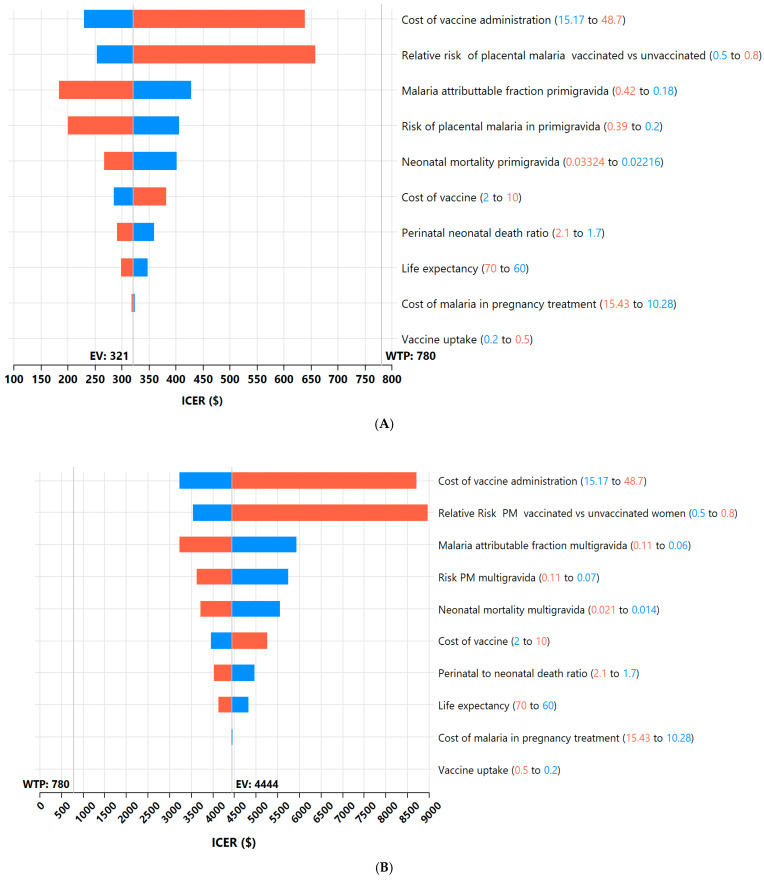
(**A**) Tornado diagram for vaccinated vs. unvaccinated primigravida. (**B**) Tornado diagram for vaccinated vs. unvaccinated multigravidae.

The tornado diagram (Figure 3A) presents the one-way sensitivity analysis evaluating how variations in key input parameters affect the ICER of vaccinating primigravidae women against placental malaria. Overall, the ICER remains relatively stable across a wide range of plausible parameter values, indicating a robust cost-effectiveness profile for this group. The cost of vaccine administration and the relative risk of placental malaria among vaccinated versus unvaccinated primigravidae are the most influential parameters, driving the greatest shifts in the ICER. Nonetheless, even at their extremes, the ICER remains well below the willingness-to-pay (WTP) threshold of $780 per DALY averted, suggesting that the vaccination maintains good value for money across these variations.

Moderate changes in the ICER arise from variations in the malaria-attributable fraction, the baseline risk of placental malaria, and neonatal mortality in primigravidae. These epidemiologic parameters influence the estimated health gains from vaccination, and their greater contribution (as compared to multigravida) reflects the higher baseline vulnerability of primigravidae and their infants to malaria-related adverse outcomes.

Less influential parameters, such as the vaccine production cost, the perinatal-to-neonatal death ratio, life expectancy, the cost of malaria treatment, and vaccine uptake, produce only minor shifts in the ICER. This indicates that even substantial uncertainty or variability in these parameters is unlikely to significantly alter the overall cost-effectiveness conclusions.

The tornado diagram (Figure 3B) shows the results of the one-way sensitivity analysis, assessing the influence of key input parameters on the ICER when vaccinating multigravidae against placental malaria. Overall, the ICER is highly sensitive to a few parameters, while most others exert relatively modest effects.

The cost of vaccine administration and the relative risk of placental malaria among vaccinated versus unvaccinated women are the two most influential drivers of the ICER. Variations in these parameters shift the ICER over the broadest range, indicating substantial uncertainty in cost-effectiveness when these values change. Moderate effects on the ICER are observed for the malaria-attributable fraction, the baseline risk of placental malaria, and neonatal mortality in multigravida women. These epidemiological parameters influence the magnitude of health benefits expected from vaccination; however, because baseline risks are already low in multigravidae, their overall impact remains limited. Other parameters, including the cost of the vaccine, the perinatal-to-neonatal death ratio, life expectancy, the cost of malaria treatment, and vaccine uptake, exert relatively small effects, resulting in only minor shifts in the ICER.

The dominance of cost-related parameters and the slight influence of epidemiological factors suggest that vaccinating multigravida women yields limited health gains due to their already low baseline risk of placental malaria and associated perinatal deaths. Consequently, even small increases in programme costs substantially worsen the cost-effectiveness profile.

**Figure 4:**
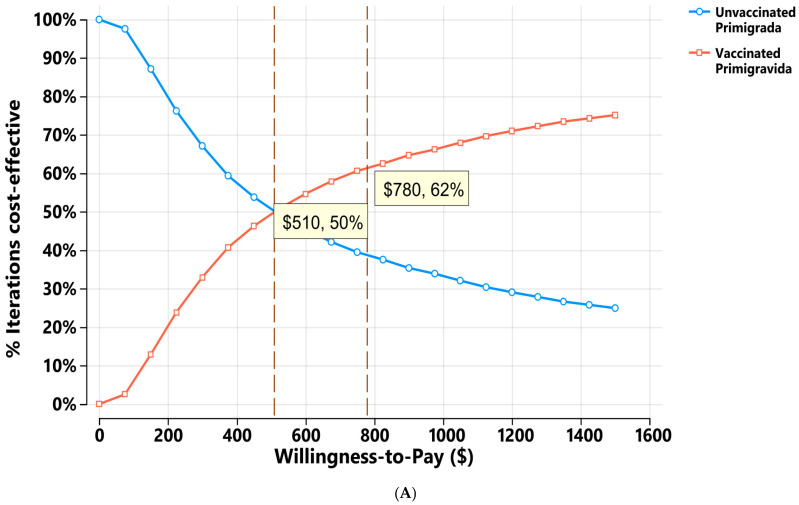

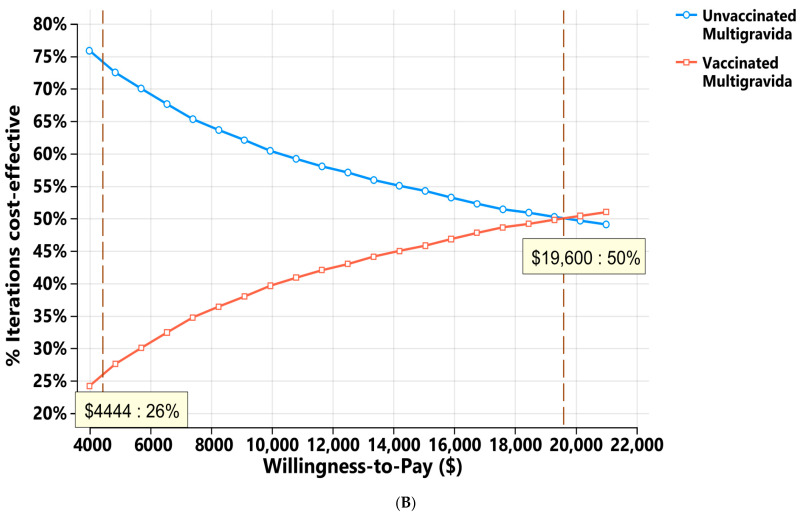
(**A**) Cost-effectiveness acceptability curve for vaccinated vs unvaccinated primigravida. (**B**) Cost-effectiveness acceptability curve for vaccinated vs unvaccinated multigravida.

The cost-effectiveness acceptability curve (Figure 4A) compares the probability that vaccination in primigravidae is cost-effective relative to no vaccination across a range of WTP thresholds. The two strategies have equal probability of being cost-effective at a WTP of approximately $510, with each having a 50% chance of being the preferred option. Beyond this point, vaccination becomes increasingly favoured. For example, at a WTP of $780, the vaccine has a 62% probability of being cost-effective. At higher WTP thresholds, vaccination clearly dominates, reaching probabilities above 70%. These results suggest that vaccinating primigravidae becomes a highly cost-effective strategy once modest WTP levels are considered.

The cost-effectiveness acceptability curve for multigravidae (Figure 4B) shows that at the baseline WTP of $4444, the vaccinating option would have only a 26% probability of being cost-effective compared with the non-vaccinating option. This indicates that at low WTP values, vaccination is very unlikely to be considered cost-effective for multigravidae. Only when the WTP exceeds $19,600 vaccinating multigravida is considered cost-effective, underscoring vaccines’ relatively low economic value in this group at commonly used thresholds.

## 4. Discussion

Reducing the risk of placental malaria in pregnancy and its associated adverse outcomes for both mothers and newborns remains a public health priority in malaria-endemic regions [51]. In this study, we evaluated the potential economic benefits of a placental malaria vaccine. Under baseline assumptions, the results indicated the vaccine was more cost-effective among primigravidae, with an ICER of $321 per perinatal DALY averted, compared to $4444 among multigravidae. Across a wide range of plausible assumptions on vaccine efficacy, cost per dose, and uptake, vaccination of primigravidae remained consistently more cost-effective than vaccination of multigravidae. These findings suggest that such a vaccine would offer greater economic value when targeted to primigravidae rather than multigravidae, owing to the higher baseline malaria risk and more severe perinatal consequences observed in first pregnancies [1]. These findings have policy and programmatic relevance, highlighting the importance of prioritising high-risk subgroups to maximise health gains and ensure the efficient allocation of limited health resources.

### 4.1. Sensitivity Analyses

The tornado diagrams illustrated that for primigravidae, the ICER remained consistently well below the WTP threshold across all parameter variations, indicating a robust cost-effectiveness profile. The ICER was most sensitive to the cost of vaccine administration and the relative risk of placental malaria among vaccinated primigravidae. Still, even at extreme values, vaccination continued to offer good value for money. Moderate influence was observed from epidemiologic parameters such as the malaria-attributable fraction, baseline placental malaria risk, and neonatal mortality, reflecting the higher underlying vulnerability of primigravida women and their infants.

In contrast, the ICER for multigravidae was far more sensitive to changes in cost-related parameters. It showed limited responsiveness to epidemiologic inputs, owing to their already low baseline risk of placental malaria and neonatal mortality. Even small increases in programme costs, such as provider training, cold chain logistics and vaccine delivery, significantly reduced the cost-effectiveness of vaccinating this group. Together, the results demonstrate that vaccinating primigravidae would provide substantial, resilient economic benefits. In contrast, cost-effectiveness among multigravidae is uncertain and highly dependent on implementation costs and other epidemiological factors.

The CEAC results indicate a clear distinction between the two groups: vaccination is far more economically favourable for primigravidae, becoming cost-effective at relatively low WTP thresholds. In contrast, vaccinating multigravidae requires substantially higher WTP levels to achieve similar cost-effectiveness. These findings highlight the strong economic case for prioritising vaccination among primigravidae, while demonstrating more limited cost-effectiveness for multigravidae under typical decision-makers’ thresholds.

### 4.2. Ethical and Equity Considerations

While these findings support the economic case for gravidity-stratified vaccination, vaccine deployment also raises ethical and equity considerations beyond efficiency alone. Malaria in pregnancy disproportionately affects young women, adolescents, and primigravidae, groups that are often socially and economically disadvantaged. Prioritising primigravidae may therefore be ethically justified under principles of equity and prioritarianism, which favour allocating resources to those at greatest risk and vulnerability [52]. However, excluding multigravidae entirely could be perceived as inequitable if it erodes trust in vaccination programmes or overlooks intersecting vulnerabilities and barriers to malaria care, particularly in hard-to-reach populations. Although this study adopts a provider perspective focused on aggregate efficiency, policy decisions on placental malaria vaccine deployment should be guided by broader deliberative processes that explicitly consider equity, acceptability, and social values. Integrating economic evidence with ethical reasoning and stakeholder perspectives is essential to ensure that vaccination strategies are not only cost-effective but also fair, acceptable, and socially legitimate in malaria-endemic settings.

### 4.3. Limitations

This study has several limitations. First, although placental malaria adversely affects both mothers and newborns, the analysis did not include maternal outcomes due to the lack of reliable data. Second, the model considered only malaria-related mortality and did not account for long-term sequelae or morbidity among newborns. Previous studies suggest that the burden of disability-related morbidity and long-term sequelae may contribute a large fraction of total DALYs, which are often under-represented when only mortality is considered [53,54]. This is indicative that our estimates are likely conservative. Future analyses that incorporate both maternal outcomes and long-term neonatal non-fatal consequences would provide a more comprehensive assessment of the health benefits and cost-effectiveness of PM vaccines. Third, the use of a single pregnancy time horizon, driven by limited longitudinal data, is another limitation. This does not capture benefits across multiple pregnancies over a woman’s reproductive life. As a result, health gains are likely underestimated and ICERs may appear less favourable. Future studies should adopt a lifetime horizon as more data become available. Fourth, the analysis does not account for waning immunity due to limited data in this Stage I evaluation. Given the limited durability of existing malaria vaccines and the potential need for booster doses, this omission may affect cost-effectiveness estimates, as protection and costs could change over time. Fifth, HPV vaccine coverage was used as a proxy for uptake. We recognise subtle differences in target population, timing, and social acceptance may affect its applicability. In addition, assumptions were required for key parameters, including vaccine efficacy and costs. These assumptions introduce uncertainty into the model estimates. To address this, we conducted scenario analyses across plausible low and high parameter estimates to test the robustness of our findings. Future evaluations incorporating empirical data on these parameters will improve the reliability of the estimates. Sixth, adopting the provider’s perspective means that only direct healthcare system costs were included, while costs borne by patients and households, such as out-of-pocket expenditures and lost productivity, were not captured. Consequently, the analysis may underestimate the full economic benefits of the vaccine, since the vaccine would reduce the need for care-seeking and associated financial burdens on households. Seventh, while the analysis indicates that prioritising vaccination for primigravidae is the most cost-effective strategy, we acknowledge that secundigravidae continue to experience a meaningful burden of placental malaria and adverse perinatal outcomes [28]. Data limitations prevented separate cost-effectiveness analysis for secundigravidae, who were therefore grouped with multigravidae, potentially underestimating the benefits of vaccination in this group. Consequently, our findings should not be interpreted as evidence that secundigravidae would not benefit from vaccination. Future studies should disaggregate gravid status to explicitly include secundigravidae, supported by more granular data, to avoid inadvertently disadvantaging this at-risk population. Finally, it is known that some women may already be infected with malaria before pregnancy [31,55]. While pre-existing malaria infection at vaccination could attenuate vaccine-induced immunity, the rate of pre-existing infection is expected to be comparable across intervention and comparator arms. Therefore, its effect is treated as a constant and does not affect the relative estimates of vaccine efficacy or downstream impacts on perinatal morbidity and mortality.

## 5. Conclusions

This economic evaluation provides early but essential evidence on the potential value of a placental malaria vaccine administered before pregnancy to women of childbearing age, in particular before their first pregnancy. By integrating regionally relevant epidemiologic, clinical, and cost data, the study highlights the vaccine’s potential to deliver favourable economic returns for primigravidae even under conservative vaccine efficacy, uptake and price assumptions. These findings reinforce the importance of continued investments in placental malaria vaccine development and the need for real-world trial data to support future economic evaluations.

## Figures and Tables

**Table 2:** Placental malaria vaccine Incremental cost-effectiveness ratios by gravidity and scenario.

Vaccinated Group	Incremental Cost-Effectiveness Ratio ($) Per Perinatal DALY ^a^ Averted		
	Baseline	Best Case Scenario ^b^	Least Favourable Scenario ^c^
Primigravidae	321	225	780
Multigravidae	4444	3148	10,598

^a^ DALY = Disability Adjusted Life Year; ^b^ Assumptions under best-case scenario (Vaccine cost = $2, vaccine uptake = 50%, vaccine efficacy = 50%); ^c^ Assumptions under worst-case scenario (Vaccine cost = $10, vaccine uptake = 20%, vaccine efficacy = 20%).

## Data Availability

All data is included in the manuscript.

## Abbreviations

The following abbreviations are used in this manuscript:

CHEERS	Consolidated Health Economic Evaluation Reporting Standards
DALYs	Disability Adjusted Life Years
GDP	Gross Domestic Product
ICER	Incremental Cost Effectiveness Ratio
IPTp-SP	Intermittent Preventive Treatment with Sulfadoxine-Pyrimethamine
IRS	Indoor Residual Spraying
ITNs	Insecticide Treated Nets
MiP	Malaria in Pregnancy
PAMVAC	Pregnancy-Associated Malaria VACcine
PM	Placental Malaria
PRIMVAC	Pregnancy-Related Immunity Against Malaria VACcine

## References

[1] Desai M., ter Kuile F.O., Nosten F., McGready R., Asamoa K., Brabin B., Newman R.D. (2007). Epidemiology and burden of malaria in pregnancy. Lancet Infect. Dis..

[2] World Health Organization (2024). World Malaria Report 2024.

[3] Satapathy P., Khatib M.N., Gaidhane S., Zahiruddin Q.S., Sharma R.K., Rustagi S., Al-Jishi J.M., Albayat H., Al Fares M.A., Garout M. (2024). Adverse pregnancy outcomes in maternal malarial infection: A systematic review and meta-analysis. New Microbes New Infect..

[4] Sundararaman S.A., Odom John A.R. (2022). Prevention of malaria in pregnancy: The threat of sulfadoxine-pyrimethamine resistance. Front. Pediatr..

[5] Kumala J., Koekemoer L.L., Coetzee M., Mzilahowa T. (2022). Intensity of insecticide resistance in the major malaria vector Anopheles funestus from Chikwawa, rural Southern Malawi. Parasit. Vectors.

[6] Darteh E.K.M., Dickson K.S., Ahinkorah B.O., Owusu B.A., Okyere J., Salihu T., Bio Bediako V., Budu E., Agbemavi W., Edjah J.O. (2021). Factors influencing the uptake of intermittent preventive treatment among pregnant women in sub-Saharan Africa: A multilevel analysis. Arch. Public Health Arch. Belg. Sante Publique.

[7] Demoze L., Adane K.C., Gizachew N., Tesfaye A.H., Yitageasu G. (2024). Utilization of insecticide-treated nets among pregnant women in East Africa: Evidence from a systematic review and meta-analysis. BMC Public Health.

[8] World Health Organization (2015). Global Technical Strategy for Malaria, 2016–2030; Global Malaria Programme.

[9] European Vaccine Initiative (2024). Advancing the Clinical Development of Placental Malaria Vaccines in the Context of Capacity Building and Use of Digital Health Technologies (ADVANCE-VAC4PM).

[10] Sirima S.B., Richert L., Chêne A., Konate A.T., Campion C., Dechavanne S., Semblat J.-P., Benhamouda N., Bahuaud M., Loulergue P. (2020). PRIMVAC vaccine adjuvanted with Alhydrogel or GLA-SE to prevent placental malaria: A first-in-human, randomised, double-blind, placebo-controlled study. Lancet Infect. Dis..

[11] Mordmüller B., Sulyok M., Egger-Adam D., Resende M., de Jongh W.A., Jensen M.H., Smedegaard H.H., Ditlev S.B., Soegaard M., Poulsen L. (2019). First-in-human, Randomized, Double-blind Clinical Trial of Differentially Adjuvanted PAMVAC, A Vaccine Candidate to Prevent Pregnancy-associated Malaria. Clin. Infect. Dis. Off. Publ. Infect. Dis. Soc. Am..

[12] Walker P.G.T., Cairns M., Slater H., Gutman J., Kayentao K., Williams J.E., Coulibaly S.O., Khairallah C., Taylor S., Meshnick S.R. (2020). Modelling the incremental benefit of introducing malaria screening strategies to antenatal care in Africa. Nat. Commun..

[13] Briggs A., Sculpher M. (1998). An introduction to Markov modelling for economic evaluation. PharmacoEconomics.

[14] Drummond M.F., Sculpher M.J., Torrance G.W., O’Brien B.J., Stoddart G.L. (2005). Methods for the Economic Evaluation of Health Care Programme.

[15] Bloom B.S., Fendrick A.M. (1996). Timing and timeliness in medical care evaluation. PharmacoEconomics.

[16] Torgerson D.J., Byford S. (2002). Economic modelling before clinical trials. BMJ.

[17] ADVANCE-VAC4PM Reducing Maternal and Children Mortality Through the Development of Safe, Effective and Affordable Vaccines Against Placental Malaria. https://www.advance-vac4pm.eu/.

[18] Moore K.A., Simpson J.A., Scoullar M.J.L., McGready R., Fowkes F.J.I. (2017). Quantification of the association between malaria in pregnancy and stillbirth: A systematic review and meta-analysis. Lancet Glob. Health.

[19] van Geertruyden J.-P., Thomas F., Erhart A., D’Alessandro U. (2004). The contribution of malaria in pregnancy to perinatal mortality. Am. J. Trop. Med. Hyg..

[20] Husereau D., Drummond M., Augustovski F., de Bekker-Grob E., Briggs A.H., Carswell C., Caulley L., Chaiyakunapruk N., Greenberg D., Loder E. (2022). Consolidated Health Economic Evaluation Reporting Standards 2022 (CHEERS 2022) Statement: Updated Reporting Guidance for Health Economic Evaluations. Value Health J. Int. Soc. Pharmacoecon. Outcomes Res..

[21] Wessel R.E., Dolatshahi S. (2023). Quantitative mechanistic model reveals key determinants of placental IgG transfer and informs prenatal immunization strategies. PLoS Comput. Biol..

[22] Healy S.A., Fried M., Richie T., Bok K., Little M., August A., Riley L., Swamy G.K., Wylie B.J., Menendez C. (2019). Malaria vaccine trials in pregnant women: An imperative without precedent. Vaccine.

[23] World Bank (2025). Life Expectancy at Birth, Total for Developing Countries in Sub-Saharan Africa.

[24] Woods B., Revill P., Sculpher M., Claxton K. (2016). Country-Level Cost-Effectiveness Thresholds: Initial Estimates and the Need for Further Research. Value Health J. Int. Soc. Pharmacoecon. Outcomes Res..

[25] Intheblack (2025). Booming Sub-Saharan Africa Set for Growth: Here’s Why.

[26] Diawara H., Healy S.A., Mwakingwe-Omari A., Issiaka D., Diallo A., Traore S., Soumbounou I.H., Gaoussou S., Zaidi I., Mahamar A. (2024). Safety and efficacy of PfSPZ Vaccine against malaria in healthy adults and women anticipating pregnancy in Mali: Two randomised, double-blind, placebo-controlled, phase 1 and 2 trials. Lancet Infect. Dis..

[27] Duffy P.E. (2022). Current approaches to malaria vaccines. Curr. Opin. Microbiol..

[28] Akinnawo A., Seyram K., Kaali E.B., Harrison S., Dosoo D., Cairns M., Asante K.P. (2022). Assessing the relationship between gravidity and placental malaria among pregnant women in a high transmission area in Ghana. Malar. J..

[29] Minwuyelet A., Yewhalaw D., Siferih M., Atenafu G. (2025). Current update on malaria in pregnancy: A systematic review. Trop. Dis. Travel Med. Vaccines.

[30] Okiring J., Olwoch P., Kakuru A., Okou J., Ochokoru H., Ochieng T.A., Kajubi R., Kamya M.R., Dorsey G., Tusting L.S. (2019). Household and maternal risk factors for malaria in pregnancy in a highly endemic area of Uganda: A prospective cohort study. Malar. J..

[31] Walker P.G.T., ter Kuile F.O., Garske T., Menendez C., Ghani A.C. (2014). Estimated risk of placental infection and low birthweight attributable to Plasmodium falciparum malaria in Africa in 2010: A modelling study. Lancet Glob. Health.

[32] Garces A., Perez W., Harrison M.S., Hwang K.S., Nolen T.L., Goldenberg R.L., Patel A.B., Hibberd P.L., Lokangaka A., Tshefu A. (2020). Association of parity with birthweight and neonatal death in five sites: The Global Network’s Maternal Newborn Health Registry study. Reprod. Health.

[33] Guyatt H.L., Snow R.W. (2001). Malaria in pregnancy as an indirect cause of infant mortality in sub-Saharan Africa. Trans. R. Soc. Trop. Med. Hyg..

[34] Goodman C.A., Coleman P.G., Mills A.J. (1999). Cost-effectiveness of malaria control in sub-Saharan Africa. Lancet.

[35] Greenwood A.M., Armstrong J.R., Byass P., Snow R.W., Greenwood B.M. (1992). Malaria chemoprophylaxis, birth weight and child survival. Trans. R. Soc. Trop. Med. Hyg..

[36] Boerma T., Campbell O.M.R., Amouzou A., Blumenberg C., Blencowe H., Moran A., Lawn J.E., Ikilezi G. (2023). Maternal mortality, stillbirths, and neonatal mortality: A transition model based on analyses of 151 countries. Lancet Glob. Health.

[37] Cirera L., Sacoor C., Meremikwu M., Ranaivo L., Manun’Ebo M.F., Arikpo D., Matavele O., Rafaralahy V., Ndombe D., Duran C.P. (2023). The economic costs of malaria in pregnancy: Evidence from four sub-Saharan countries. Gates Open Res..

[38] Tembo I., Kalele J.C., Simwinga B., Mgemezulu T., Mwanza T., Kazonga E., Mudenda S., Mutemwa R. (2024). Prevalence and predictors of malaria in pregnant women in Sub-Saharan Africa: A systematic review and meta-analysis. Int. J. Community Med. Public Health.

[39] LaMontagne D.S., Cernuschi T., Yakubu A., Bloem P., Watson-Jones D., Kim J.J., Bundy D.A.P., de Silva N., Horton S., Jamison D.T., Patton G.C. (2017). School-Based Delivery of Vaccines to 5- to 19-Year Olds. Child and Adolescent Health and Development.

[40] Kwizera A., Ntasumumuyange D., Small M., Rulisa S., Moscovitz A.N., Magriples U. (2021). Assessment of perinatal outcomes of pregnant women with severe versus simple malaria. PLoS ONE.

[41] Asgedom Y.S., Kebede T.M., Seifu B.L., Mare K.U., Asmare Z.A., Asebe H.A., Kase B.F., Shibeshi A.H., Tebeje T.M., Sabo K.G. (2024). Human papillomavirus vaccination uptake and determinant factors among adolescent schoolgirls in sub-Saharan Africa: A systematic review and meta-analysis. Hum. Vaccines Immunother..

[42] World Bank Group MetaData. https://databank.worldbank.org/metadataglossary/world-development-indicators/series/NY.GDP.DEFL.ZS.

[43] Baral R., Levin A., Odero C., Pecenka C., Bawa J.T., Antwi-Agyei K.O., Amponsa-Achaino K., Chisema M.N., Jalango R.E., Mkisi R. (2023). Cost of introducing and delivering RTS,S/AS01 malaria vaccine within the malaria vaccine implementation program. Vaccine.

[44] UNICEF (2025). Malaria Vaccine Questions and Answers.

[45] World Health Organization (2023). WHO Recommends R21/Matrix-M Vaccine for Malaria Prevention in Updated Advice on Immunization.

[46] Diawara H., Bocoum F.Y., Dicko A., Levin A., Lee C., Koita F., Ouédraogo J.B., Guissou R., Yabré S., Traoré S. (2023). Cost of introducing and delivering malaria vaccine (RTS,S/AS01E) in areas of seasonal malaria transmission, Mali and Burkina Faso. BMJ Glob. Health.

[47] Slavkovsky R., Callen E., Pecenka C., Mvundura M. (2024). Costs of human papillomavirus vaccine delivery in low- and middle-income countries: A systematic review. Vaccine.

[48] Gray A.M., Clarke P., Wolstenhome J.L., Wordsworth S. (2011). Applied Methods of Cost-Effectiveness Analysis in Healthcare.

[49] Fenwick E., O’Brien B.J., Briggs A. (2004). Cost-effectiveness acceptability curves—facts, fallacies and frequently asked questions. Health Econ..

[50] D'Alessio F., Honkpehedji J., Sirima S., Gamain B., Morten N., Ndam N., Mordmüller B., Dicko A., Adechina R., Dossou A. (2026). Immunization strategies to prevent malaria in pregnancy-A multistakeholder workshop. Vaccine.

[51] World Health Organization (2025). Maternal Health promotion and protection, and prevention of complications. WHO Recommendations on Maternal Health Guidelines Approved by the WHO Guidelines Review Committee [Internet].

[52] Cookson R., Mirelman A.J., Griffin S., Asaria M., Dawkins B., Norheim O.F., Verguet S., Culyer A.J. (2017). Using Cost-Effectiveness Analysis to Address Health Equity Concerns. Value Health J. Int. Soc. Pharmacoecon. Outcomes Res..

[53] Bauserman M., Conroy A.L., North K., Patterson J., Bose C., Meshnick S. (2019). An Overview of Malaria in Pregnancy. Semin. Perinatol..

[54] Schiess N., Villabona-Rueda A., Cottier K.E., Huether K., Chipeta J., Stins M.F. (2020). Pathophysiology and neurologic sequelae of cerebral malaria. Malar. J..

[55] Berry I., Walker P., Tagbor H., Bojang K., Coulibaly S.O., Kayentao K., Williams J., Oduro A., Milligan P., Chandramohan D. (2018). Seasonal Dynamics of Malaria in Pregnancy in West Africa: Evidence for Carriage of Infections Acquired Before Pregnancy Until First Contact with Antenatal Care. Am. J. Trop. Med. Hyg..

